# ﻿Two new *Inosperma* (Inocybaceae) species with unexpected muscarine contents from tropical China

**DOI:** 10.3897/mycokeys.85.71957

**Published:** 2021-12-15

**Authors:** Lun-Sha Deng, Rui Kang, Nian-Kai Zeng, Wen-Jie Yu, Cheng Chang, Fei Xu, Wang-Qiu Deng, Liang-Liang Qi, Yu-Ling Zhou, Yu-Guang Fan

**Affiliations:** 1 Key Laboratory of Tropical Translational Medicine of Ministry of Education, Transgenic Laboratory, Tropical Environment and Health Laboratory, College of Pharmacy, Hainan Medical University, Haikou 571199, China Hainan Medical University Haikou China; 2 Hainan Institute for Food Control, Haikou 570314, China Hainan Institute for Food Control Haikou China; 3 Jilin Provincial Joint Key Laboratory of Changbai Mountain Biocoenosis and Biodiversity, Changbai Mountain Academy of Sciences, Yanbian 133613, China Jilin Provincial Joint Key Laboratory of Changbai Mountain Biocoenosis and Biodiversity, Changbai Mountain Academy of Sciences Yanbian China; 4 Physical and Chemical Department, Ningxia Hui Autonomous Region Center for Disease Control and Prevention, Yinchuan 750004, China Physical and Chemical Department, Ningxia Hui Autonomous Region Center for Disease Control and Prevention Yinchuan China; 5 State Key Laboratory of Applied Microbiology Southern China, Guangdong Provincial Key Laboratory of Microbial Culture Collection and Application, Guangdong Institute of Microbiology, Guangdong Academy of Sciences, Guangzhou 510070, China Guangdong Institute of Microbiology, Guangdong Academy of Sciences Guangzhou China; 6 Microbiology Research Institute, Guangxi Academy of Agriculture Sciences, Nanning 530007, China Microbiology Research Institute, Guangxi Academy of Agriculture Sciences Haikou China

**Keywords:** Agaricales, muscarine, new species, phylogeny, taxonomy

## Abstract

An accurate identification of poisonous mushrooms and the confirmation of the toxins involved are both of great importance in the treatment of mushroom poisoning incidents. In recent years, cases of mushroom poisoning by *Inosperma* spp. have been repeatedly reported from tropical Asia. It is urgent to know the real species diversity of *Inosperma* in this region. In the present study, we proposed two new *Inosperma* species from tropical Asia, namely *I.muscarium* and *I.hainanense*. They were described based on morphology and multilocus phylogeny. Detailed descriptions, color photographs and the discussion with other closely related species of the two new taxa were provided. In addition, a comprehensive muscarine determination of these two new species using ultrahigh performance liquid chromatography tandem mass spectrometry (UPLC-MS/MS) approach has been performed. Results showed that these two species were muscarine positive, with a content of 16.03 ± 1.23 g/kg in *I.muscarium* and a content of 11.87 ± 3.02 g/kg in *I.hainanense*, much higher than the known species *I.virosum*. Recovery of muscarine ranged from 93.45% to 97.25%, and the average recovery is 95.56%.

## ﻿Introduction

Muscarine C_9_H_20_NO_2_^+^, CAS number: 300–54–9, is a toxic alkaloid found in Inocybaceae, *Clitocybe* and several other mushroom genera (Patocka et al. 2021). The ingestion of muscarine-containing mushrooms would cause diaphoresis, salivation, urination, nausea, vomiting, gastrointestinal effects and muscular cramp, and fatal muscarinic syndromes like miosis, bronchoconstriction, and bradycardias in humans (Wilson 1947; Lurie et al. 2009; Chandrasekharan et al. 2020; Latha et al. 2020; Patocka et al. 2021), or even death (Pauli et al. 2005; Işıloğlu et al. 2009; Zosel et al. 2015). Many species of Inocybaceae are known to contain muscarine (Malone et al. 1962), especially in *Inocybesensu stricto*, and *Pseudosperma* (Kosentka et al. 2013; Matheny et al. 2020). *Inosperma*, a genus in Inocybaceae, is supposed to contain only a small number of muscarine positive species (Kosentka et al. 2013). However, mushroom poisoning events caused by *Inosperma* species were repeatedly reported from tropical Asia in recent years (Chandrasekharan et al. 2020; Li et al. 2021; Parnmen et al. 2021). Accordingly, it is urgent to enrich the knowledge of species diversity of the genus and to detect their muscarine toxin contents in tropical Asia.

*Inosperma* was erected as a subgenus of *Inocybe* with *Inocybecalamistrata* (Fr.) Gillet as type (Kühner 1980), and is now treated as genus rank (Matheny et al. 2020). Members in this genus are characterized by small to medium-sized basidiomata, rimose to scaly pileus, often rubescent context, phaseoliform to subglobose basidiospores, thin-walled cheilocystidia, lack of pleurocystidia, and often with distinctive odors. *Inosperma* species are widespread and there are seventy-one taxa documented globally (http://www.indexfungorum.org, retrieved 7 Oct. 2021). The tropical elements of *Inosperma* comprise several recently described, and still a few undescribed taxa, which were divided into two separate Old World tropical clades (Kropp et al. 2013; Matheny et al. 2020; Aïgnon et al. 2021; Deng et al. 2021). Interestingly, most of the taxa from Old World tropical clade 1 were mainly distributed in western Africa (Matheny et al. 2020; Aïgnon et al. 2021), and species in Old World tropical clade 2 were mainly from tropical Asia (Deng et al. 2021).

During our field works around the tropical China, two new *Inosperma* species were discovered. The present study aims to describe these two new tropical species using a combined data of morphology and phylogeny, and to determine their muscarine contents, in order to provide an accurate data for the prevention and clinical treatment of potential *Inosperma* poisoning accidents.

## ﻿Materials and methods

### ﻿Research area and specimens sampling

Our collections were made from *Castanopsis* dominated forests in Hainan, Guangdong Provinces, and Guangxi Zhuang Autonomous Region of China, with a tropical or subtropical climate. Specimens were photographed in the field using a digital camera and then described soon after collection. The specimens were dried through an electronic drier at 45 °C overnight, and were then preserved in plastic bags and sealed. After study, dried specimens were deposited in the Fungal Herbarium of Hainan Medical University (FHMU), Haikou City, Hainan Province of China, or in the Fungarium of Guangdong Institute of Microbiology (GDGM), Guangzhou, China.

### ﻿Morphological study

Marcoscopic features were made from field notes and photographs. Color notations follow Kornerup and Wanscher (1978). Microscopic characters from dried materials mounted in KOH (5%) or mixed with Congo Red (1%) solution were observed with a microscope and photographed using a digital camera. Randomly selected twenty basidiospores and ten basidia for each specimen, the length and width of each basidiospore and basidium were measured, excluding the apiculus and sterigmata respectively (Kobayashi 2009). Numbers in square brackets [n/m/p] represent “n” basidiospores measured from “m” basidiomata of “p” specimens (Zhang et al. 2019). The dimensions of basidiospores and Q values are expressed as (a) b–c (d), “a” and “d” denote extreme values (“a” < 5^th^ percentile; “d” > 95^th^ percentile), while the ranges “b–c” means 5^th^ to 95^th^ percentile values. The quotient Q = length/width ratio for individual basidiospore, and Q_m_ means the average of Q values (Dramani et al. 2020).

### ﻿DNA extraction, PCR and sequencing

Genomic DNA was extracted from dried specimens using the NuClean Plant Genomic DNA kit (ComWin Biotech, Beijing). The following primers were used: ITS1F/ITS4 for ITS (Gardes and Bruns 1993), LR0R/LR7 for LSU (Vilgalys and Herster 1990), bRPB2-6F/bRPB2-7.1R for *rpb2* (Matheny 2005). The volume of polymerase chain reaction (PCR) mixture solution was 25 μL, containing 9.5 μL dd H_2_O, 12.5 μL 2×Taq Plus MasterMix (Dye), 1 μL of each primer, and 1 μL of template DNA. PCR conditions for ITS, LSU and *rpb2* followed Wang et al. (2021), that the conditions of PCR for three different gene regions are all the same as denaturation at 95 °C for 1 min at first, then followed by 35 cycles of denaturation at 95 °C for 30 s, annealing at 52 °C for 1 min, extension at 72 °C for 1 min, and a final extension at 72 °C for 8 min. Afterwards, the products of amplifications were sent to the Beijing Genomics Institute for purification and sequenced as soon as possible.

### ﻿Analysis of sequence data

Sequences in this study were prepared and compared with closely related *Inosperma* sequences that were retrieved from GenBank (https://www.ncbi.nlm.nih.gov/) through BLAST tool (https://blast.ncbi.nlm.nih.gov/Blast.cgi) or literature survey (Larsson et al. 2009; Kropp et al. 2013; Horak et al. 2015; Nasser et al. 2017; Bau and Fan 2018; Matheny and Kudzma 2019; Matheny et al. 2020; Deng et al. 2021; Aïgnon et al. 2021; Cervini et al. 2021; Bandini et al. 2021). Then sequences from three genes were aligned respectively using MAFFT online service (https://mafft.cbrc.jp/alignment/server/) (Katoh et al. 2019) and were edited by BioEdit version 7.0.9.0 (Hall 1999). Two taxa in *Auritella* (*A.hispida* and *A.spiculosa*) were served as outgroups (Matheny et al. 2020). MrModeltest v2.3 was used to select the best-fit model for each gene partition for Bayes analysis (Nylander 2004). The datasets of each locus were combined in MEGA 5.02 (Tamura 2011). Maximum likelihood (ML) was inferred under partitioned models using W-IQ-TREE Web Service (http://iqtree.cibiv.univie.ac.at/), and the ultrafast bootstrapping was done with 1000 replicates (Trifinopoulos et al. 2016). Bayesian analysis was performed in MrBayes v.3.2.7a (Ronquist et al. 2012).

### ﻿Muscarine toxin detection

Methods for sample preparation and analysis through UPLC-MS/MS were followed by Xu et al. (2020) with some modifications. Dried samples were ground to a fine power respectively, to 20 mg of each homogenised portion, 2 mL methanol-water solution (5:95 v/v) was added. The extraction was vortexed in a vortex mixer for 30 min, the mixture was further extracted by using an ultrasonic bath for another 30 min, and centrifuged for 5 min with 10000 rpm speed. Total supernatant was collected, using 0.22 μm organic filter membrane to filtrate for UPLC-MS/MS analysis and diluted with methanol-water (5:95, v/v) when necessary. The blank sample used here was *Lentinulaedodes*. The optimal MS parameters and product ion confirmation settings followed Xu et al. (2020), while the chromatographic column we used was ACQUITY UPLC BEH Amide (2.1 mm × 100 mm, 1.7 µm). The muscarine content was estimated in the mushroom extract by using standard muscarine (Sigma-Aldrich, Chemical purity ≥ 98%). The analytical results are reported as Mean ± SD g/kg, where Mean is the average content of muscarine in the mushroom from each experimental species, and SD represents its standard deviation.

## ﻿Results

### ﻿Phylogenetic inference

**Figure 1.**
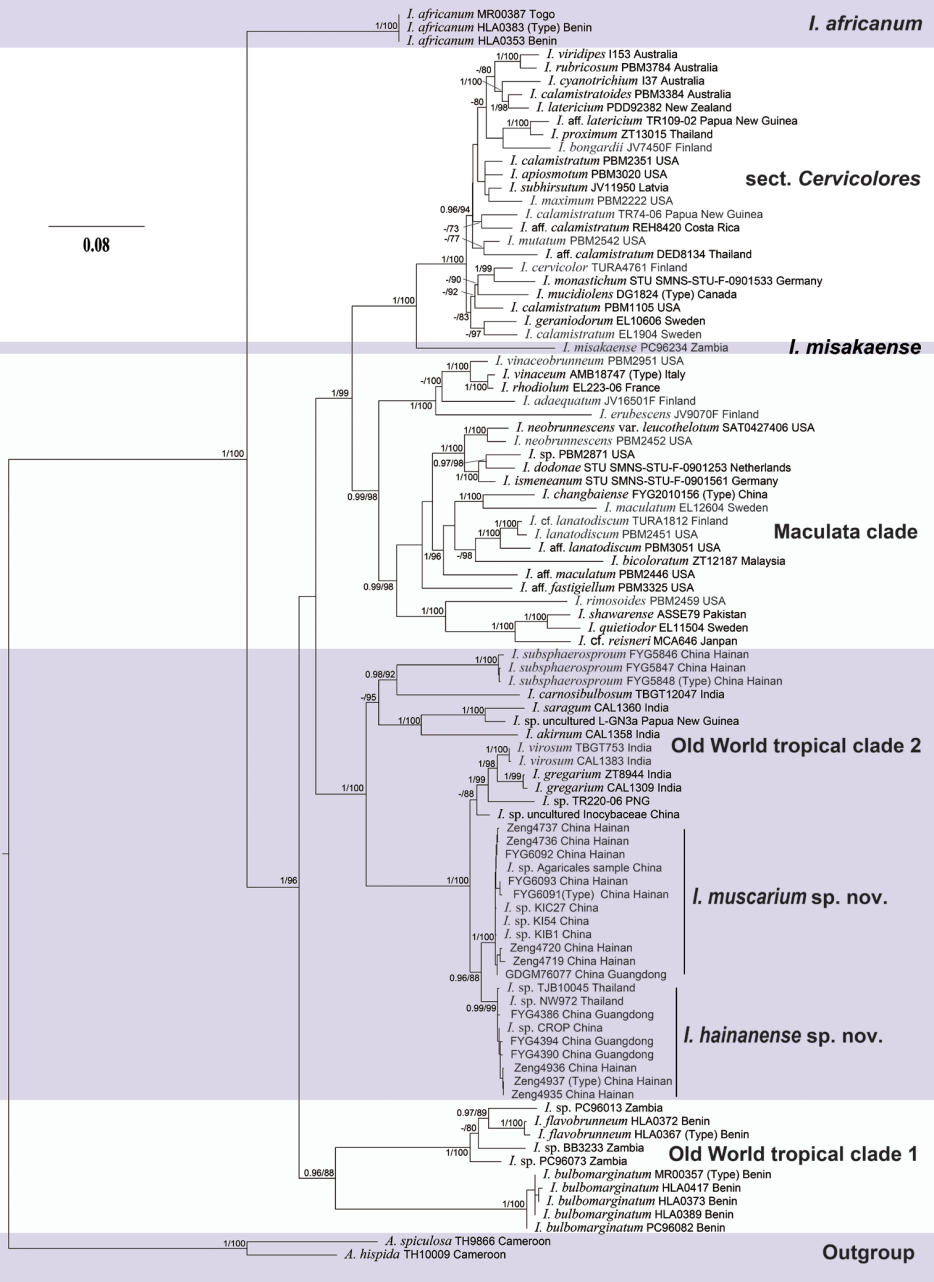
Phylogram generated by Bayesian Inference (BI) analyses based on sequences of a combined data set from nuclear genes (rDNA-ITS, nrLSU, and *rpb2*), rooted with *Auritellahispida* and *A.spiculosa.* Bayesian Inference posterior probabilities (BI-PP) ≥0.95 and ML bootstrap proportions (ML-BP) ≥70 are represented as BI-PP/ML-BP. *I.muscarium* sp. nov. and *I.hainanense* sp. nov. are two newly described taxa.

The final multilocus dataset (Table 1) includes 94 taxa and 3130 characters, and 37 new sequences (14 ITS, 12 LSU and 11 *rpb2*) were generated in this study and then submitted to GenBank. The alignment was deposited in TreeBase (28515). The best-fit models for each gene selected by MrModelGUI are GTR+I+G equally. The Maximum likelihood (ML) and Bayesian analyses for the combined dataset provide a best scoring tree is shown in Fig. 1. Three ectomycorrhizal samples (KIC27, KI54, and KIB1) and an environmental sample grouped together with eight specimens of *I.muscarium* with significant support (BP = 100%, PP = 1). In addition, two specimens (TJB10045 and NW972) from Thailand and an environmental sample (CROP denovo 1461) from China grouped together with six specimens of *I.hainanense* with high support (BP = 99%, PP = 0.99). The two new *Inosperma* species formed separate lineages and were sister with significant support (BP = 88%, PP = 0.96) to each other. These two new species formed a subclade in the Old World tropical clade 2. The subclade was sister to *I.virosum* (K.B. Vrinda, C.K. Pradeep, A.V. Joseph & T.K. Abraham ex C.K. Pradeep, K.B. Vrinda & Matheny) Matheny & Esteve-Rav., *I.gregarium* (K.P.D. Latha & Manimohan) Matheny & Esteve-Rav., and an undescribed specimen *I.* sp. (TR220-06) from Papua New Guinea with full support (BP = 100%, PP = 1).

**Table 1.** Taxon sampling information and DNA sequences used for phylogenetic analyses

Taxa	Collection number/Herbaium	Locality	GenBank accession number			Reference
			ITS	LSU	*rpb2*	
*Auritellahispida*	TH10009	Cameroon	KT378203	KT378207	KT378215	Matheny et al. (2020)
*Auritellaspiculosa*	TH9866	Cameroon	KT378204	KT378206	KT378214	Matheny et al. (2020)
*Inospermaadaequatum*	JV16501F	Finland	–	AY380364	AY333771	Matheny et al. (2020)
Inospermaaff.lanatodiscum	PBM3051	USA	JQ801401	JN975026	JQ846485	Pradeep et al. (2016)
Inospermaaff.calamistratum	DED8134	Thailand	GQ892983	GQ892937	–	Pradeep et al. (2016)
Inospermaaff.calamistratum	REH8420	Costa Rica	JQ801390	JN975018	JQ846471	Pradeep et al. (2016)
Inospermaaff.fastigiellum	PBM3325	USA	JQ801399	JQ815419	JQ846477	Pradeep et al. (2016)
Inospermaaff.latericium	TR109-02	Papua New Guinea	JQ801405	JN975023	JQ846487	Pradeep et al. (2016)
*Inospermaaff.maculatum*	PBM2446	USA	DQ241778	AY745700	EU569863	Pradeep et al. (2016)
*Inospermaafricanum*	MR00387	Togo	MN096189	MN097881	MT770739	Aïgnon et al. (2021)
*Inospermaafricanum*	HLA0383 (Type)	Benin	MT534298	MT560733	–	Aïgnon et al. (2021)
*Inospermaafricanum*	HLA0353	Benin	MT534299	–	–	Aïgnon et al. (2021)
*Inospermaakirnum*	CAL1358	India	KY440085	KY549115	KY553236	Matheny et al. (2020)
*Inospermaapiosmotum*	PBM3020	USA	JQ801385	JN975021	JQ846463	Matheny et al. (2020)
*Inospermabicoloratum*	ZT12187	Malaysia	GQ892984	GQ892938	JQ846464	Pradeep et al. (2016)
*Inospermabongardii*	JV7450F	Finland	–	EU555448	–	Pradeep et al. (2016)
*Inospermabulbomarginatum*	MR00357 (Type)	Benin	MN096190	MN097882	MN200775	Aïgnon et al. (2021)
*Inospermabulbomarginatum*	HLA0417	Benin	MT534300	MT560734	–	Aïgnon et al. (2021)
*Inospermabulbomarginatum*	HLA0373	Benin	MT534301	–	–	Aïgnon et al. (2021)
*Inospermabulbomarginatum*	HLA0389	Benin	MT534302	–	–	Aïgnon et al. (2021)
*Inospermabulbomarginatum*	PC96082	Benin	JQ801412	JN975027	–	Aïgnon et al. (2021)
*Inospermacalamistratoides*	PBM3384	Australia	JQ801393	JQ815415	KJ729949	Pradeep et al. (2016)
*Inospermacalamistratum*	PBM1105	USA	JQ801386	JQ815409	JQ846466	Pradeep et al. (2016)
*Inospermacalamistratum*	EL1904	Sweden	AM882938	AM882938	–	Pradeep et al. (2016)
*Inospermacalamistratum*	PBM2351	USA	–	AY380368	AY333764	Pradeep et al. (2016)
*Inospermacalamistratum*	TR74-06	Papua New Guinea	JQ801391	JN975020	JQ846472	Pradeep et al. (2016)
*Inospermacarnosibulbosum*	TBGT12047	India	KT329448	KT329454	KT329443	Pradeep et al. (2016)
*Inospermacervicolor*	TURA4761	Finland	JQ801395	JQ815417	JQ846474	Pradeep et al. (2016)
Inospermacf.lanatodiscum	TURA1812	Finland	JQ408763	JQ319694	JQ846484	Pradeep et al. (2016)
Inospermacf.reisneri	MCA646	Japan	–	EU555463	–	Pradeep et al. (2016)
*Inospermachangbaiense*	FYG2010156 (Type)	China	MH047251	MG844976	MT086755	Bau and Fan (2018)
*Inospermacyanotrichium*	I37	Australia	JQ801396	JN975033	JQ846476	Pradeep et al. (2016)
*Inospermadodonae*	SMNS-STU-F-0901253	Netherlands	MW647615	–	–	Bandini et al. (2021)
*Inospermaerubescens*	JV9070F	Finland		EU569846	–	Pradeep et al. (2016)
*Inospermaflavobrunneum*	HLA0372	Benin	MT534290	MT536756	–	Aïgnon et al. (2021)
*Inospermaflavobrunneum*	HLA0367 (Type)	Benin	MN096199	MT536754	–	Aïgnon et al. (2021)
*Inospermageraniodorum*	EL10606	Sweden	FN550945	FN550945	–	Pradeep et al. (2016)
*Inospermagregarium*	ZT8944	India	–	EU600903	EU600902	Pradeep et al. (2016)
*Inospermagregarium*	CAL1309	India	KX852305	KX852306	KX852307	Latha and Manimohan. (2016)
***Inospermahainanense***	**Zeng4936**	**China**	**MZ374069**	**MZ374760**	**MZ388103**	**The present study**
***Inospermahainanense***	**Zeng4937 (Type)**	**China**	**MZ374070**	**MZ374761**	**MZ388104**	**The present study**
***Inospermahainanense***	**Zeng4935**	**China**	**MZ374071**	**MZ374762**	**MZ388105**	**The present study**
***Inospermahainanense***	**FYG4386**	**China**	**MZ374072**	–	–	**The present study**
***Inospermahainanense***	**FYG4390**	**China**	**MZ374073**	**MZ374763**	–	**The present study**
***Inospermahainanense***	**FYG4394**	**China**	**MZ374068**	–	–	**The present study**
*Inospermaismeneanum*	STU:SMNS-STU-F-0901561	Germany	MW647625	–	–	Bandini et al. (2021)
*Inospermalanatodiscum*	PBM2451	USA	JQ408759	JQ319690	JQ846483	Pradeep et al. (2016)
*Inospermalatericium*	PDD92382	New Zealand	GU233367	GU233413	–	Pradeep et al. (2016)
*Inospermamaculatum*	EL12604	Sweden	AM882964	AM882964	–	Pradeep et al. (2016)
*Inospermamaximum*	PBM2222	USA		EU569854	–	Pradeep et al. (2016)
*Inospermamisakaense*	PC96234	Zambia	JQ801409	EU569875	AY333767	Pradeep et al. (2016)
*Inospermamonastichum*	STU:SMNS-STU-F-0901533	Germany	MW647631	–	–	Bandini et al. (2021)
*Inospermamucidiolens*	DG1824 (Type)	Canada	HQ201339	HQ201340	–	Pradeep et al. (2016)
***Inospermamuscarium***	**Zeng4720**	**China**	**MZ373978**	**MZ373988**	**MZ388089**	**The present study**
***Inospermamuscarium***	**Zeng4736**	**China**	**MZ373979**	**MZ373989**	**MZ388090**	**The present study**
***Inospermamuscarium***	**Zeng4737**	**China**	**MZ373980**	–	**MZ388091**	**The present study**
***Inospermamuscarium***	**Zeng4719**	**China**	**MZ373981**	**MZ373990**	**MZ388092**	**The present study**
***Inospermamuscarium***	**FYG6091 (Type)**	**China**	**MZ373982**	**MZ373991**	**MZ388093**	**The present study**
***Inospermamuscarium***	**FYG6092**	**China**	**MZ373983**	**MZ373992**	**MZ388094**	**The present study**
***Inospermamuscarium***	**FYG6093**	**China**	**MZ373984**	**MZ373993**	**MZ388095**	**The present study**
***Inospermamuscarium***	**GDGM76077**	**China**	**MZ520549**	**MZ520550**	**MZ542730**	**The present study**
*Inospermaneobrunnescens*	PBM2452	USA	–	EU569868	EU569867	Pradeep et al. (2016)
Inospermaneobrunnescensvar.leucothelotum	SAT0427406	USA	JQ801411	JN975025	JQ846489	Pradeep et al. (2016)
*Inospermaproximum*	ZT13015	Thailand	EU600839	EU600840		Matheny et al. (2020)
*Inospermaquietiodor*	EL11504	Sweden	AM882960	AM882960		Pradeep et al. (2016)
*Inospermarhodiolum*	EL223-06	France	FJ904175	FJ904175		Pradeep et al. (2016)
*Inospermarimosoides*	PBM2459	USA	DQ404391	AY702014	DQ385884	Pradeep et al. (2016)
*Inospermarubricosum*	PBM3784	Australia	KP308817	KP170990	KM406230	Pradeep et al. (2016)
*Inospermasaragum*	CAL1360	India	KY440103	KY549133	KY553249	Latha and Manimohan (2017)
*Inospermashawarense*	ASSE79	Pakistan	KY616964	KY616966		Naseer et al. (2018)
*Inosperma* sp.	PBM2871	USA	HQ201348	HQ201348	JQ846475	Pradeep et al. (2016)
*Inosperma* sp.	BB3233	Zambia	JQ801415	EU600885		Pradeep et al. (2016)
*Inosperma* sp.	L-GN3a	Papua New Guinea	JX316732	JX316732		Pradeep et al. (2016)
*Inosperma* sp.	TJB10045	Thailand	KT600658	KT600659	KT600660	Pradeep et al. (2016)
*Inosperma* sp.	TR22006	Papua New Guinea	JQ801416	JN975017	JQ846496	Pradeep et al. (2016)
*Inosperma* sp.		China	LS983441			Unpublished
*Inosperma* sp.	CROP	China	MF532817			Unpublished
*Inosperma* sp.		China	LS975930			Unpublished
*Inosperma* sp.	NW972	Thailand	MN492637			Unpublished
*Inosperma* sp.	KIB1	China	JX456867			Unpublished
*Inosperma* sp.	KIC27	China	JX456949			Unpublished
*Inosperma* sp.	KI54	China	JX456860			Unpublished
*Inosperma* sp.	PC96013	Zambia	JQ801383	EU600883	EU600882	Pradeep et al. (2016)
*Inosperma* sp.	PC96073	Zambia	JQ801417	EU600870	EU600869	Pradeep et al. (2016)
*Inospermasubhirsutum*	JV11950	Latvia		EU555452	AY333763	Pradeep et al. (2016)
*Inospermasubsphaerosproum*	FYG5848 (Type)	China	MW403825	MW397171	MW404237	Deng et al. (2021)
*Inospermasubsphaerosproum*	FYG5847	China	MW403826	MW397172	MW404238	Deng et al. (2021)
*Inospermasubsphaerosproum*	FYG5846	China	MW403827	MW397173	MW404239	Deng et al. (2021)
*Inospermavinaceobrunneum*	PBM2951	USA		HQ201353	JQ846478	Pradeep et al. (2016)
*Inospermavinaceum*	AMB18747	Italy	MW561108	MW561120		Cervini et al. (2021)
*Inospermaviridipes*	I153	Australia	KP641646	KP171095	KM656139	Pradeep et al. (2016)
*Inospermavirosum*	TBGT753	India	KT329452	KT329458	KT329446	Pradeep et al. (2016)
*Inospermavirosum*	CAL1383	India	KY440108	KY549138	KY553253	Latha and Manimohan (2017)

## ﻿Taxonomy

### ﻿ Inosperma muscarium

Taxon classificationFungiAgaricalesInocybaceae

Y.G. Fan, L.S. Deng, W.J. Yu & N.K. Zeng sp. nov.

0B930071-9800-5B82-8872-66B1A6353BA0

MB840527

Figures 2 , 3

#### Etymology.

“*muscarium*” refers to its high content of muscarine.

**Holotype.** China, Hainan Province, Ledong Li Autonomous County, Yinggeling substation of Hainan Tropical Rainforest National Park, under *Castanopsis* forest, at 19°1'20"N, 109°23'33"E, alt. 550 m, 26 April 2021, FYG6091 (FHMU3162), GenBank accession number: ITS (MZ373982); LSU (MZ373991) and *rpb2* (MZ388093).

#### Diagnosis.

Basidiomata small to medium-sized. Pileus rimulose to rimose with an indistinct umbo, lamellae rather crowded. Basidiospores smooth, enlongate ellipsoid to ellipsoid. Cheilocystidia clavate. Under *Castanopsis* forest. Differs from *I.hainanense* by its more robust habit, elongate basidiospores, and narrower cheilocystidia.

**Figure 2.**
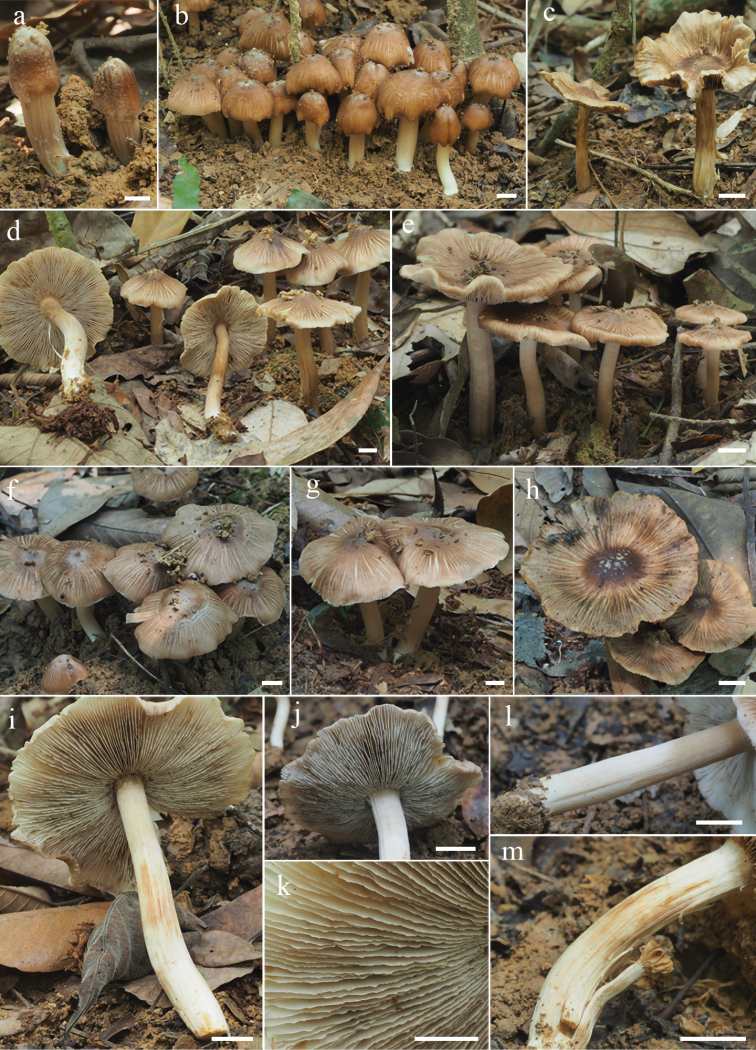
Basidiomata of *Inospermamuscarium***a–e** basidiomata **f–h** rimose to rimulose pileus **i** lamellae **j–k** lamellae edge **l–m** stipe surface. **a–b, d, f–g, i–m** FHMU3162 (holotype) **c, e** FYG6092 (FHMU3163) **h** FYG6093 (FHMU3164). Scale bars: 10 mm (**a–m**). Photos by Y.-G. Fan.

**Figure 3.**
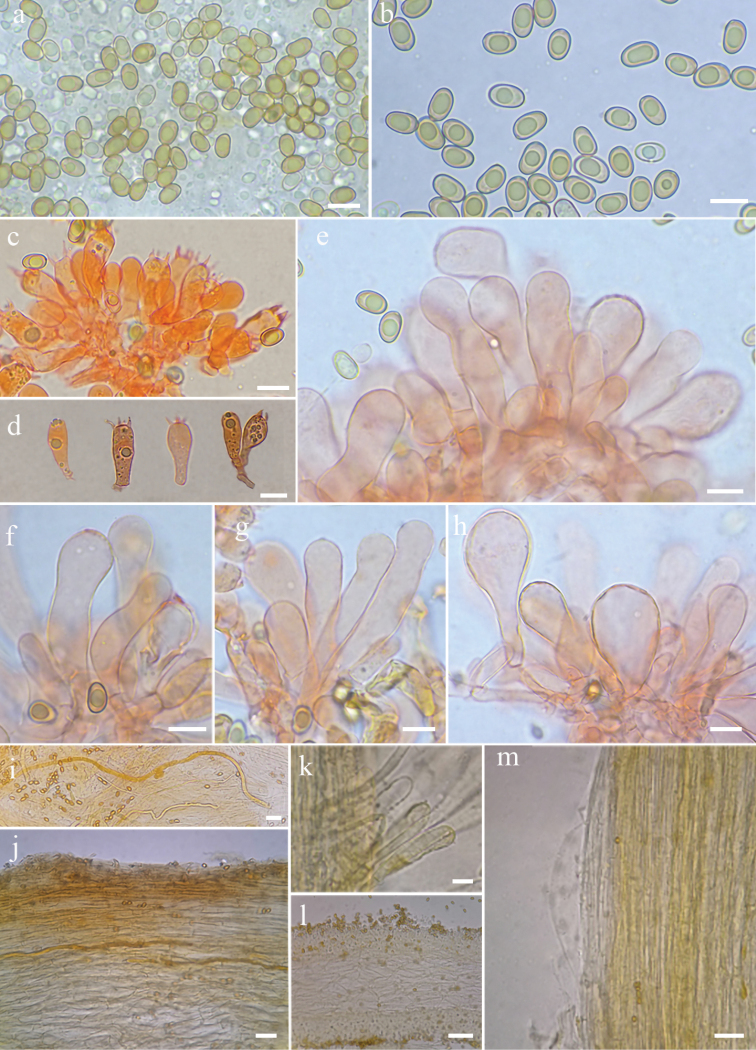
Microscopic features of *Inospermamuscarium* (FHMU3162, holotype) **a–b** basidiospores **c–d** basidia **e–h** cheilocystidia in clusters **i** oleiferous hyphae **j** pileipellis and pileal trama **k** terminal hyphae at the stipe apex **l** hymenophoral trama **m** stipitipellis and stipe trama. Scale bars: 10 μm (**a–m**). Photos by L.-S. Deng

#### Basidiomata.

small to medium-sized. Pileus 25–60 mm diam., conical convex to convex when young, becoming broadly convex to plano-convex with a small indistinct umbo when mature, margin slightly incurved when young, becoming somewhat reflexed with age. Surface dry, smooth with distinct ivory white (5A1) veil layer around the disc when young, then appressed with indistinct veil remnants, fibrillose-rimulose elsewhere, margin usually strongly rimose with age; yellowish brown (5D8) to chocolate brown (5E8) around the center and on the fibrils, yellowish brown (5C6) elsewhere, yellowish brown (6C6) to slightly dark brown (6E7) all over the basidiomata when overmatured. Lamellae rather crowded, adnexed, initially pure white to pale off-white (4B1), becoming grayish white (5B1) to yellowish white (4A2), dirty yellow (4A3) to yellowish brown (5B4) when overmatured, 1.5–3 mm wide, edge fimbriate, faint serrate to somewhat wavy. Stipe 35–72 × 3–8 mm, central, solid, terete, equal with a slightly swollen apex and base; with sparse fibrils at apex, longitudinally fibrillose downwards the stipe, with white tomentose hyphae at the base; initially white (5A1) to cream white(3A2), yellowish (4A3) or brownish (5A3) with age, brown (5B6) to dark brown (5C5) when old. Context solid, fleshy in pileus, 0.5–1 mm thick at mid-radius, 1.5–4.5 mm under the umbo, white to ivory white (5A1) at first, becoming brownish white (5B2); fibrillose and striate in the stipe, white to yellowish (4A2) or flesh color (4B3). Odor fungoid, slightly grassy or mild.

#### Basidiospores.

[180/9/9] 8–10(11) × 5–6 (6.5) μm, Q = (1.15)1.42–1.86(2.00), Q_m_=1.63, mostly ellipsoid to enlongate ellipsoid, occasionally sub-phaseoliform, smooth, thick-walled, yellowish, apiculus small, indistinct, with a spherical to ellipsoid yellowish brown oil-droplet inside. Basidia 17–24 × 7–9 μm, clavate to broadly clavate, obtuse at apex, slightly tapering towards the base, 4-spored, sterigmata 2–4 μm in length, thin-walled, hyaline or pale yellow, with oily drops in various sizes with age. Pleurocystidia none. Lamella edge sterile. Cheilocystidia 36–50 × 9–14 μm, abundant and crowded, mostly clavate, broadly clavate to enlongate-clavate, rarely balloon-shaped, apices rounded to obtuse, or occasionally subcapitate, thin- to slightly thick-walled, septate, often constricted at septa, colorless to yellowish, sometimes with golden yellow inclusions. Hymenophoral trama 75–108 μm thick, sub-regular, colorless to yellowish, composed of thin-walled, smooth, cylindric to mostly inflated, hyphae 12–25 μm wide, somewhat constricted at the both ends of per hyphae. Pileipellis a cutis, sub-regular, composed of thin-walled, brown to yellowish brown, cylindrical, slightly encrusted hyphae 4–10 μm wide. Pileal trama colorless, regular to subregular, hyphae 12–25 μm wide. Stipitipellis a cutis, regularly arranged, occasionally with small clusters of terminal cheilocystidoid cells at the stipe apex, cheilocystidoid cells 31–47 × 9–10 μm, rare, clavate to enlongate clavate, hyaline or pale yellow, thin- to slightly thick-walled, some with golden yellow inclusions. Caulocystidia not observed. Oleiferous hyphae 4–13 μm wide, scattered in pileus and stipe tramal tissue, yellow or bright golden yellow, smooth, often bent, sometimes diverticulate. Clamp connections present, common in all tissues.

#### Habitat.

Gregarious in clusters, usually scattered with numerous clusters under *Castanopsis* forest, late March to August in tropical China.

#### Known distribution.

China (Hainan, Guangdong, Guangxi), Thailand.

#### Additional materials examined.

China. Hainan Province, Ledong Li Autonomous County, Yinggeling substation of Hainan Tropical Rainforest National Forest Park, under *Castanopsis* forest, 13 August 2020, N.K. Zeng, Zeng4720 (FHMU3158); Same location, under *Castanopsis* forest, 14 August 2020, N.K. Zeng Zeng4736 (FHMU3159); Zeng4737 (FHMU3160), Same location, 26 April 2021, Y.G. Fan, L.S. Deng & Q.Q. Chen, FYG6092 (FHMU3163); FYG6093 (FHMU3164); FYG6094 (FHMU3173); Guangdong Province, Yangchun City, Gangmei Town, Lunshui Village, under *Castanopsis* forest, 29 March 2019, W.Y. Huang, GDGM76077; Guangxi Zhuang Autonomous Region: Wuzhou City, Cangwu Country, Wangfu Town, 23°40'28"N, 111°29'6"E, alt. 30 m, Under *Castanopsis* dominated forest, 29 May 2021, L.L. Qi, WSW10286, (FHMU3174).

### ﻿ Inosperma hainanense

Taxon classificationFungiAgaricalesInocybaceae

Y.G. Fan, L.S. Deng, W.J. Yu & N.K. Zeng sp. nov.

BAF0240A-9583-5E18-B038-28AD3D315F33

MB840528

Figures 4 , 5

#### Etymology.

“*hainanense*” refers to the its type locality.

**Holotype.** China, Hainan Province, Changjiang Li Autonomous County, Bawangling substation of Hainan Tropical Rainforest National Park, under *Castanopsis* dominated forest, at 19°7'12.43"N, 109°7'6.29"E, alt. 630 m, 2 September, 2020, N.K. Zeng, Zeng4937 (FHMU3166), GenBank accession number: ITS (MZ374070); LSU (MZ374761) and *rpb2* (MZ388104).

#### Diagnosis.

Distinguishes from *I.muscarium* by its slender basidiomata, ellipsoid to ovoid basidiospores, and mostly vesiculose cheilocystidia.

**Figure 4.**
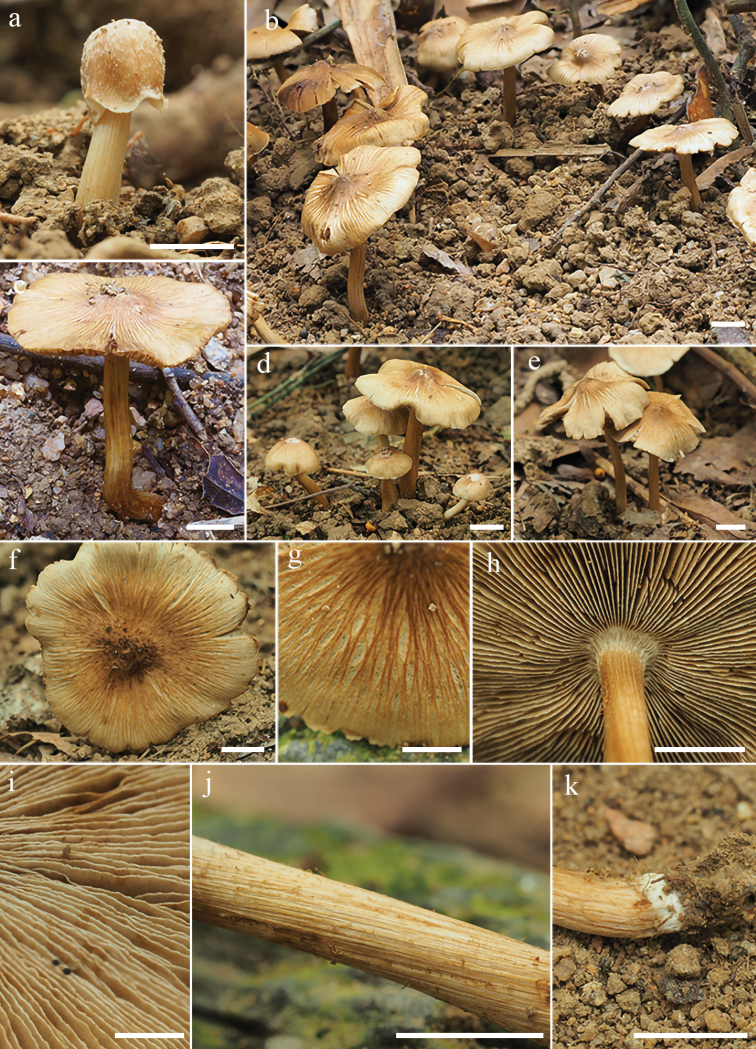
Basidiomata of *Inospermahainanense***a–e** basidiomata **f–g** rimose to rimulose pileus **h** lamellae **i** lamellae edge **j–k** stipe surface. **c** FHMU3166 (holotype) **a–b, d–g, i–k** FHMU6511 **h** FHMU3168. Scale bars: 10 mm (**a–k**). **a–b, d–k**: photos by L.-S. Deng; **c**: photos by N.-K. Zeng

**Figure 5.**
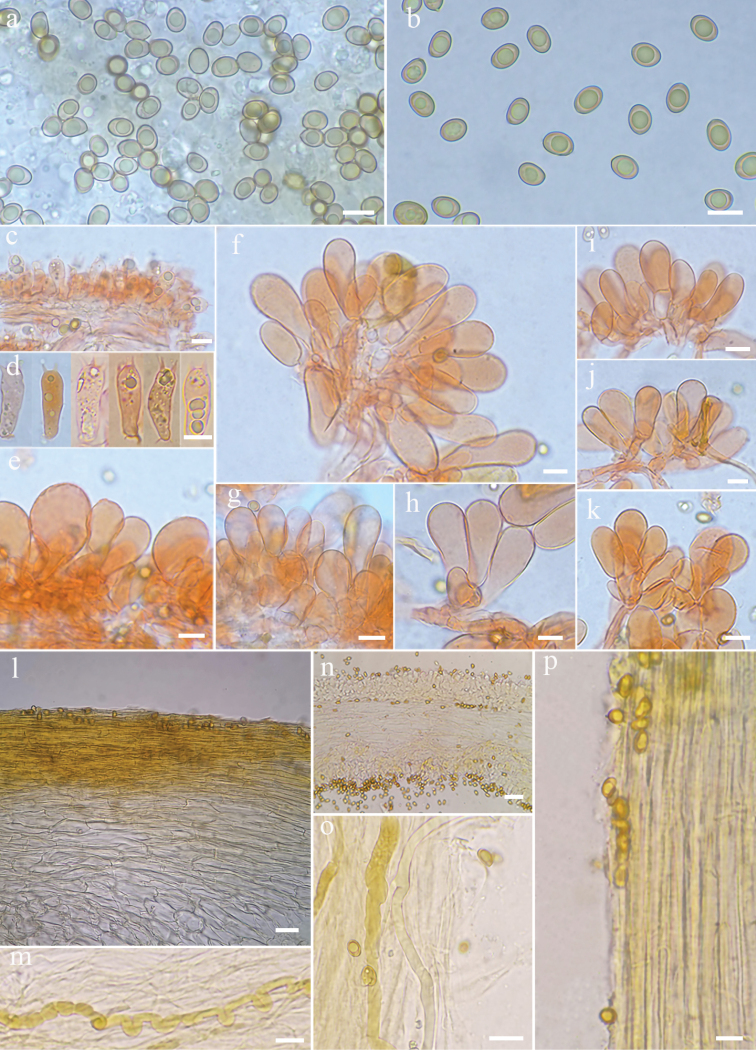
Microscopic features of *Inospermahainanense* (FHMU3166, holotype) **a–b** basidiospores **c–d** basidia **e–k** cheilocystidia in clusters **l** pileipellis and pileal trama **n** hymenophoral trama **m, o** oleiferous hyphae **p** stipitipellis and stipe trama. Scale bars: 10 μm (**a–k**). Photos by L.-S. Deng

#### Basidiomata.

small to medium-sized. Pileus 25–53 mm diam., conical to convex at young age, becoming applanate to uplifted with age, with a broad to subacute umbo, margin initially decurved, straight to somewhat wavy when mature; surface dry, smooth when young, fibrillose-rimulose elsewhere, strongly rimose towards the margin with age; chocolate brown (5D8) to somewhat dark brown (5F7) around the disc, straw yellow (4A6) to yellowish brown (4B5) elsewhere, background pallid to cream white (4B1), becoming brown (5B4) to dark brown (5C6) with age; Lamellae rather crowded, adnexed, initially ivory white (5A1) to grayish white (5B2), becoming dirty yellowish (5B5) to brownish (5C7) when matured, completely brown (5D6) after drying, 2–3 mm in width, edge fimbriate, slightly serrate. Stipe 40–72 × 3–5 mm, central, nearly terete, equal with a slightly swollen apex, base somewhat swollen; nearly smooth and longitudinally striate all over the stipe; initially ivory (5A1) to yellowish white (5A2) at the upper half, yellowish to brownish (4B5) downwards, becoming uniformly yellowish brown (4B7) to brown (4C7) with age. Context solid, fleshy in pileus, white to grayish white (4B1), pale brown under the umbo (4B2), 1–2 mm thick at mid-radius, 4–5 mm thick under the umbo, fibrillose in stipe, pallid to yellowish (4A2) or brownish (4B2), striate, shiny. Odor indistinct or slightly acid.

#### Basidiospores.

[180/9/9] 8–9(10.5) × 5–7 μm, Q = (1.18)1.28–1.64 (1.78), Q_m_ = 1.43, mostly ellipsoid to ovoid, occasionally subphaseoliform, smooth, slightly thick-walled, brown to yellowish brown, apiculus small, indistinct, with a spherical to ellipsoid yellowish brown oil-droplet. Basidia 21–28 × 6–9 μm, clavate, often obtuse at apex, slightly tapered towards the base, thin-walled, 4-spored, sometimes 2-spored, sterigmata 4–6 μm in length, with spherical yellowish brown to golden yellow brown oily inclusions. Pleurocystidia absent. Lamella edge sterile. Cheilocystidia 34–55 × 15–25 μm, abundant and crowded, mostly obovoid to balloon-shaped, occasionally broadly clavate, rarely enlongate-clavate, thin- to slightly thick-walled (up to 1 μm thick); often rounded or slightly obtuse at apex, colorless to pale yellow, sometimes with golden yellow pigments. Hymenophoral trama 75–138 μm thick, sub-regular, hyaline to slightly yellow, composed of cylindric to inflated hyphae 20–33 μm wide, slightly constricted at septa. Pileipellis a cutis, hyphae 2.5–10 μm wide, thin-walled, pale yellow to yellowish brown, cylindrical, sometimes slightly encrusted. Pileal trama regular to subregular, hyphae 12–30 μm wide, thin-walled, colorless. Stipitipellis a cutis, regularly arranged, walls yellowish to bright yellow. Oleiferous hyphae 2.5–10 μm wide, commonly scattered in pileus and stipe tramal tissues, straw yellow or bright golden yellow, smooth, often bent or diverticulate. Clamp connections observed in all tissues.

#### Habitat.

Scattered or gregarious in small clusters under *Castanopsis* dominated forest, June to September in tropical China.

#### Known distribution.

China (Hainan, Guangdong).

#### Additional materials examined.

China. Hainan Province, Wuzhishan City, Maoyang Town, Maoyang Village, 11 August 2021, Y.G. Fan & L.S. Deng, FYG6440 (FHMU6513); Ganshiling Provincial Nature Reserve, L.S. Deng & Y.G. Fan, DLS0043 (FHMU6512); Changjiang Li Autonomous County, Bawangling substation of Hainan Tropical Rainforest National Park, under *Castanopsis* dominated forest, 2 September 2020, N.K. Zeng, Zeng4936 (FHMU3165); Zeng4935 (FHMU3167); Guangdong Province, Guangzhou City, Tianluhu Forest Park, 2 June 2019, Y.G. Fan & W.J. Yu, FYG4386 (FHMU3168); Shaoguan City, Danxiashan Nature Reserve, 4 June 2019, Y.G. Fan & W.J. Yu, FYG4388 (FHMU3175); 4390 (FHMU3169); FYG4394 (FHMU3170).

##### ﻿Muscarine detection

Representative chromatograms of muscarine were shown in Fig. 8. The muscarine toxin content was confirmed by linear equation according to the analysis of UPLC-MS/MS, it was found that both of the two new species contained muscarine toxin, and the content of *Inospermamuscarium* was 16.03 ± 1.23 g/kg while *I.hainanense* was 11.87 ± 3.02 g/kg. Muscarine was identified by comparing retention time (1.22 min) and relative deviation (0.82%) in the allowable relative range of 25 % base on the qualitative analysis. The calibration curve for muscarine generated during the validation was *y* = 2083.17 *x*–209.297 (*r* = 0.9988) for muscarine concentration in the range of 2–200 ng/mL (*y* represents the peak area, and *x* is muscarine concentration, *r* is correlation coefficient). Recovery of muscarine ranged from 93.45% to 97.25%, and the average recovery was 95.56%.

## ﻿Discussion

### ﻿New species delimitation

**Figure 6.**
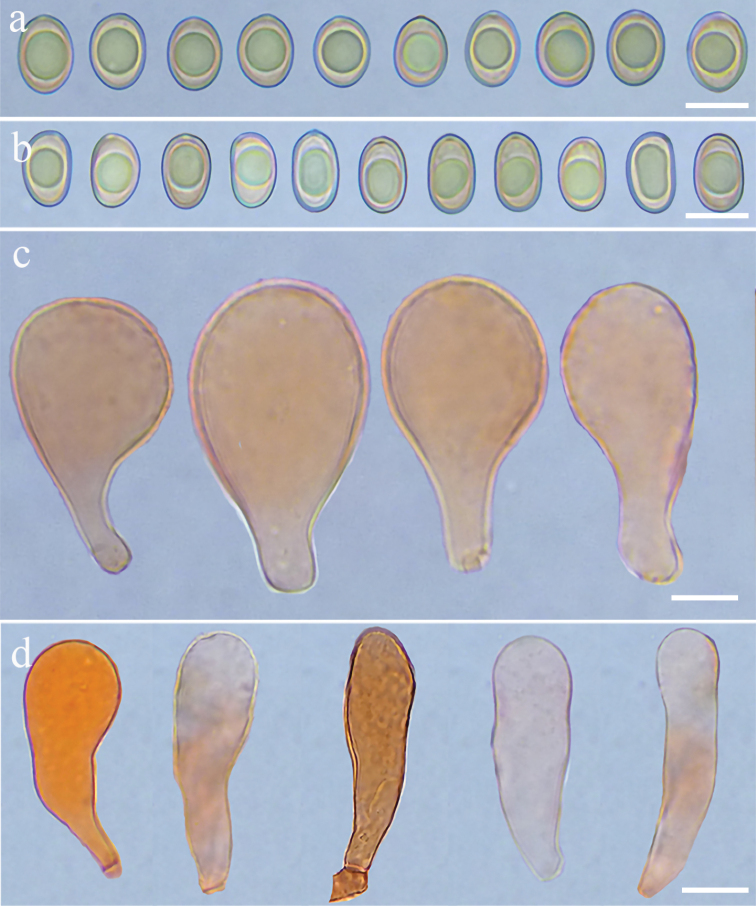
The comparisons of the two new species in their outline of basidiospores and cheilocystidia shape **a, c** basidiospores and cheilocystidia of *I.hainanense* (FHMU3162, holotype); **b, d** Basidiospores and cheilocystidia of *I.muscarium* (FHMU3166, holotype). Scale bars: 10 μm (**a–d**). Photos by L.-S. Deng

**Figure 7.**
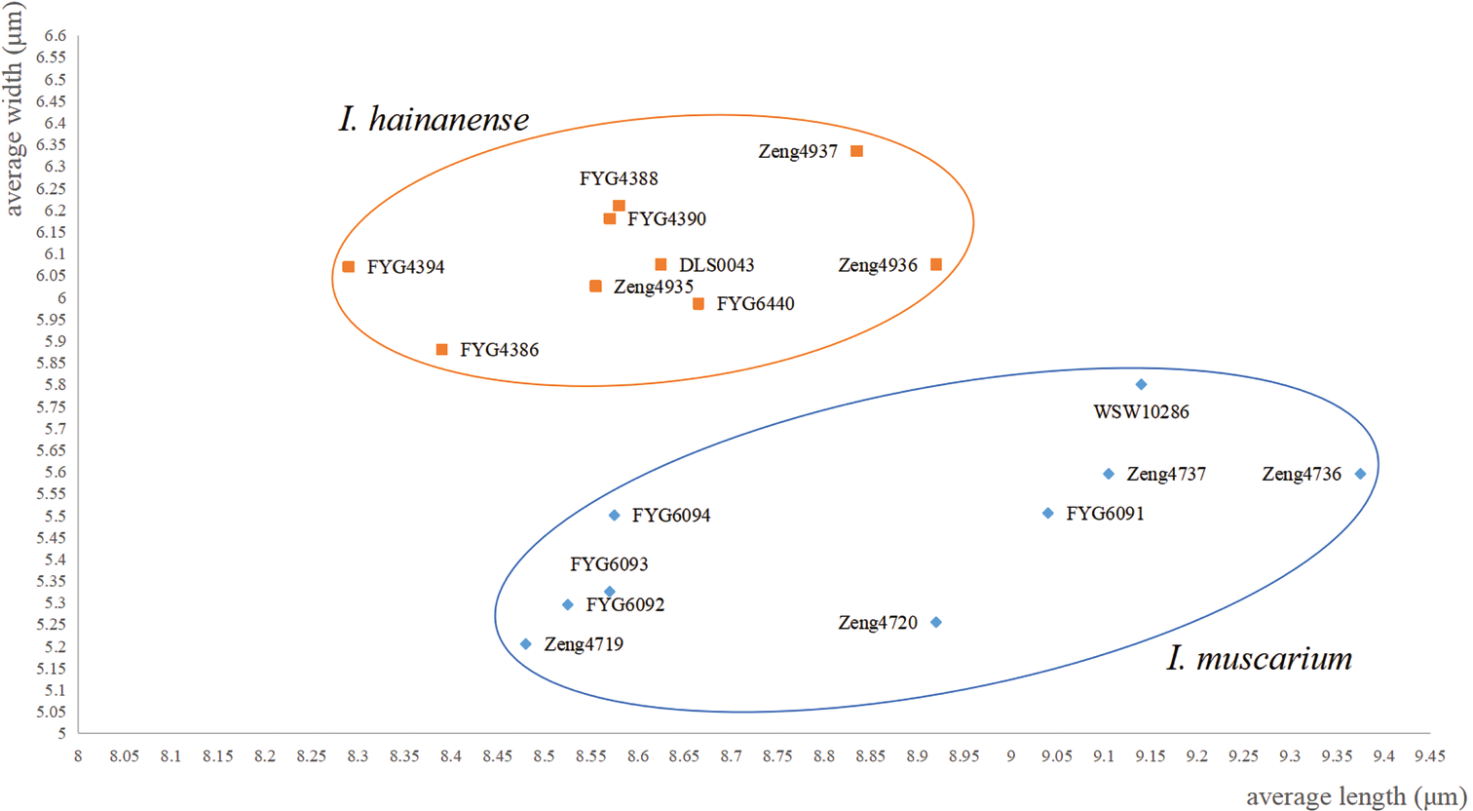
The comparisons of the two new species in their dimensions of basidiospores.

The phylogenetic results place both the two new species in the Old World tropical clade 2 in genus *Inosperma* (Kropp et al. 2013; Pradeep et al. 2016; Deng et al. 2021), and they are sister to each other with significant support (BP = 88%, PP = 0.96). Morphologically, they share yellowish brown pileus, longitudinally striate stipe, crowded lamellae, and elliptic basidiospores. It is really difficult to distinguish the two new species by their macromorphology, in spite of the fact that *I.hainanense* has a relatively more slender habit, more finely rimulose in pileus, and a smoother stipe surface. However, they could be easily distinguished by their outlines of basidiospores and cheilocystidia. As is shown in Figs 6–7, *I.muscarium* has more elongated basidiospores in outline, as well as narrower cheilocystidia (*I.muscarium*: 36–50 × 9–14 μm; *I.hainanense*: 34–55 × 15–25 μm).

**Figure 8.**
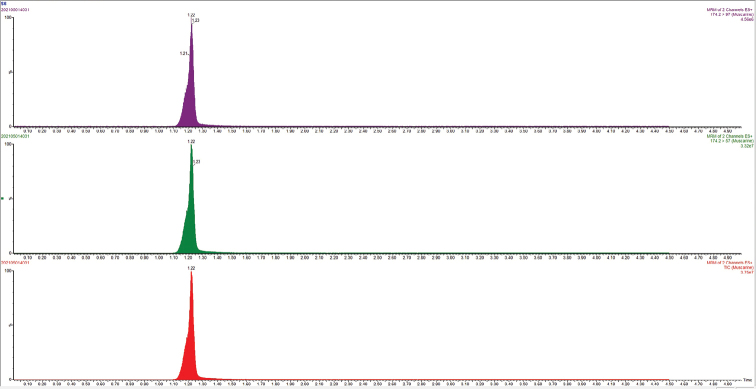
Representative chromatograms of muscarine.

In Old World tropical clade 2, *I.gregarium* and *I.virosum*, both of which described from India, formed a sister lineage with the two new species. They also share fibrillose-rimose pileus, longitudinally striate stipe, crowded lamellae, and elliptic basidiospores (Vrinda et al. 1996; Latha and Manimohan 2016). However, *I.gregarium* differs from the two new species by its smaller basidiospores (7–8.5 × 5–5.5 μm, Q = 1.3–1.8, Q_m_ = 1.6), versiform and longer cheilocystidia (24–60 × 16–24 µm), the presence of caulocystidia, and an association with Dipterocarpaceae trees (Latha and Manimohan 2016). *Inospermavirosum* differs in having smaller basidiospores (6.5–8.5 × 5–6 µm, Q = 1.3–1.6, Q_m_ = 1.4), and an association also with Dipterocarpaceae trees (Vrinda et al. 1996; Latha and Manimohan 2017). The remaining species in this subgrouping resemble the two new species to some extent; however, they have appressed-scaly or appressed-fibrillose pileus and different phylogenetic positions (Latha and Manimohan 2017).

There are eight described species in Old World tropical clade 2 so far, three of which were described from China in Fagaceae forest (Deng et al. 2021), and the rest five species were all described from India under Dipterocarpaceae forest or among ginger plants (Pradeep et al. 2016; Latha and Manimohan 2017). By our current knowledge, members in this subgrouping usually have medium-sized basidiomata, gregarious habit, appressed-scaly or fibrillose-rimose pileus, rather crowded lamellae, longitudinally striate stipe, non-changing context, subglobose to elliptic basidiospores, and the lack of distinctive odors (Pradeep et al. 2016; Latha and Manimohan 2017; Deng et al. 2021).

### ﻿Muscarine toxin in Inosperma

The compound muscarine was initially isolated and identified from *Amanitamuscaria* with the content at about 0.0003% of the fresh weight (Spoerke and Rumack 1994). However, muscarine was more commonly found in Inocybaceae and *Clitocybe* spp. with significant concentrations reached the highest record of 1.6%. (Lurie et al. 2009). Many Inocybaceae species were well known to contain muscarine (Peredy et al. 2014; Patocka et al. 2021), and various methods have been used to detect this toxin in the past years (Fahrig 1920; Eugster 1957; Brown et al. 1962; Robbers 1964; Kosentka et al. 2013; Latha et al. 2020). Five *Inosperma* species were reported as muscarine positive, including *I.cervicolor* (Pers.) Matheny & Esteve-Rav., *I.erubescens* (A. Blytt) Matheny & Esteve-Rav., *I.maculatum* (Boud.) Matheny & Esteve-Rav., *I.vinaceobrunneum* (Matheny, Ovrebo & Kudzma) Haelew. and *I.virosum* (K.B. Vrinda, C.K. Pradeep, A.V. Joseph & T.K. Abraham ex C.K. Pradeep, K.B. Vrinda & Matheny) Matheny & Esteve-Rav. (Kosentka et al. 2013; Latha et al. 2020). In addition, *I.carnosibulbosum* (C.K. Pradeep & Matheny) Matheny & Esteve-Rav., a species described from India, is probably a muscarine positive species due to a recent report of poisonous case (Chandrasekharan et al. 2020). Among these muscarine positive species in *Inosperma*, *I.virosum* described from India, is more extensively studied in toxin detection, toxicity in vitro using NCM460 colon epithelial cell line, toxic effects in vivo and pharmacokinetics of muscarine (Latha et al. 2020). The muscarine content of *I.virosum* is 270 or 300 mg/kg reported by separate studies (Sailatha et al. 2014; Latha et al. 2020).

Surprisingly, of the two new species we assayed, both of them have a high content of muscarine that is about 30 to 50 times higher than *I.virosum* (Sailatha et al. 2014; Latha et al. 2020). For humans, a lethal dose of muscarine is estimated from 40 mg to 495 mg (Pauli et al. 2005). Based on the muscarine concentrations of between 0.1% to 0.33% (dry weight) in Inocybaceae spp., a single mushroom can be lethal (Puschner 2018; Patocka et al. 2021). Consequently, the two new species proposed by the present study were considered to be more dangerous when mistakenly ingested by humans. In particular, for *I.muscarium*, a species often with a medium-sized basidiomata, a gregarious, large, discrete clusters habitat, and the lack of aposematic coloration make it extremely easily collected by local people as an edible mushroom. The publicity and education of the two new species were essential to prevent mushroom poisoning from tropical areas where they distributed.

The accurate identification of poisonous mushrooms and the knowledge of toxin type and contents are crucial for the treatment of mushroom poisoning patients (Li et al. 2021). However, species identification can usually be difficult for doctors when faced with mushroom-poisoned patients, mainly because of the insufficient identification data of wild poisoning mushrooms (Hall et al. 1987). Our present study provides detailed knowledge for a better prevention of potential *Inosperma* poisoning from tropical Asia.

## Supplementary Material

XML Treatment for Inosperma muscarium

XML Treatment for Inosperma hainanense
